# An Atypical Presentation of Lemierre’s Syndrome: Complicated by Thrombotic Thrombocytopenic Purpura

**DOI:** 10.7759/cureus.12728

**Published:** 2021-01-15

**Authors:** Bryan Vera Nieves, Geoffrey Lindblad, Javas Gupta, Jessica Hughes, Andres Rivero

**Affiliations:** 1 Infectious Disease, Aventura Hospital and Medical Center, Miami, USA; 2 Radiology, Aventura Hospital and Medical Center, Miami, USA; 3 Internal Medicine, Aventura Hospital and Medical Center, Miami, USA

**Keywords:** lemierre's syndrome, fusobacterium necrophorum, thrombotic thrombocytopenic purpura, thrombocytopenia, lemierre, lemierre's

## Abstract

Lemierre’s syndrome is an oropharyngeal infection complicated by septic thrombophlebitis of the internal jugular vein, bacteremia, and septic emboli. It mainly occurs in immunocompetent individuals and was first reported in the early 1900s by physician Andre Lemierre. A 23-year-old male presented to our institution with sore throat, difficulty swallowing, left-sided ear pain, nausea, vomiting, subjective fevers, general malaise, right-sided rib pain, and anorexia. Complete blood cell count and metabolic panels revealed severe thrombocytopenia, mild anemia, acute kidney injury, and hyperbilirubinemia. Blood cultures grew *Fusobacterium necrophorum*. Ultrasound and computed tomography scan of the neck revealed thrombosis of the left internal jugular vein. ADAMTS13 activity was later reported to be markedly decreased at less than 2%, confirming a diagnosis of thrombotic thrombocytopenic purpura.

## Introduction

Lemierre’s syndrome is a rare condition characterized by septic thrombophlebitis of the internal jugular vein and bacteremia. It usually occurs following a recent oropharyngeal infection and is frequently complicated by septic emboli [1-4]. While thrombocytopenia has been reported in patients with Lemierre’s syndrome, thrombotic thrombocytopenic purpura (TTP) as the cause of the thrombocytopenia in patients with Lemierre’s syndrome has not been described in literature to our knowledge [5]. We report a case of Lemierre’s syndrome complicated by TTP.

## Case presentation

**Figure 1:**
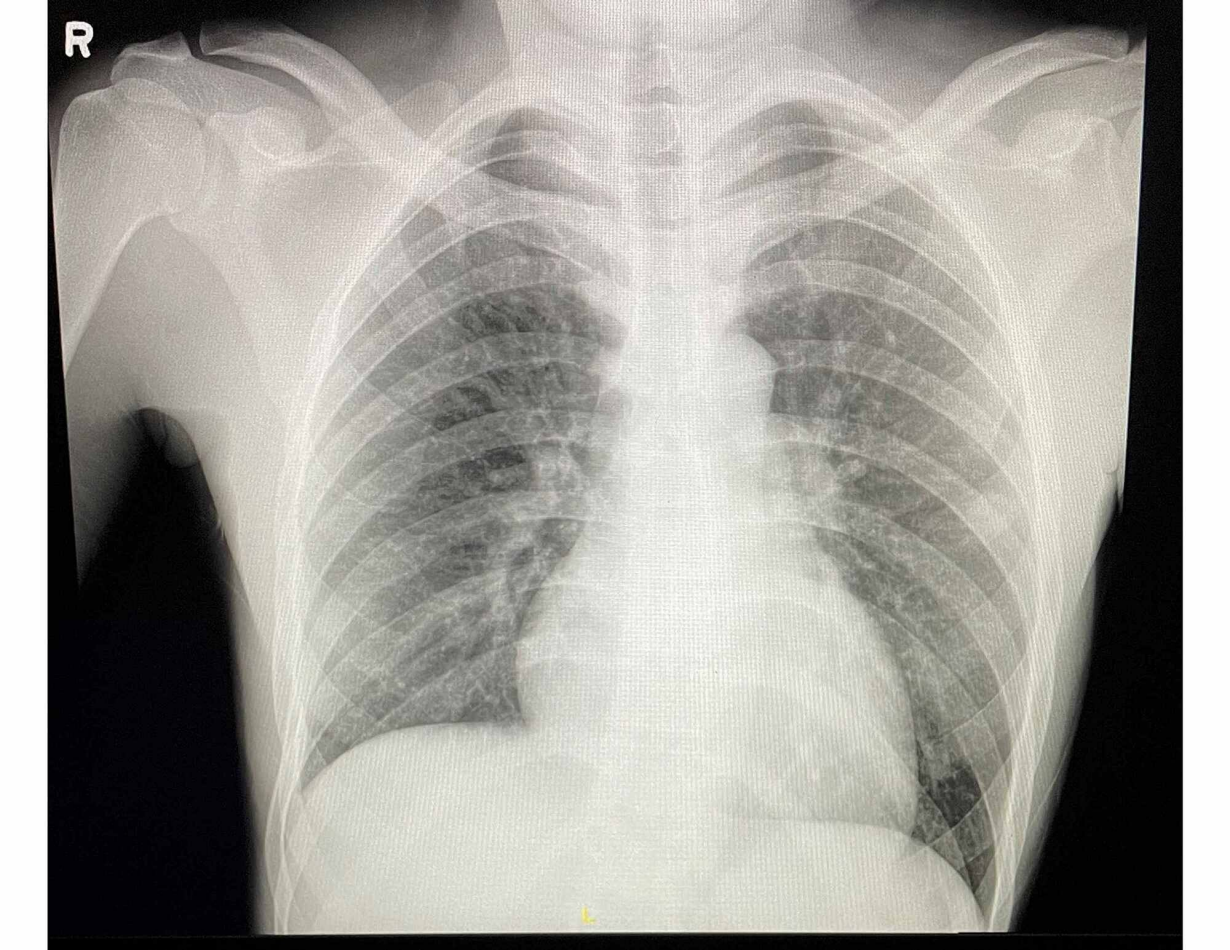
Chest radiograph showing scattered, non-calcified pulmonary nodules.

Our patient is a 23-year-old male who was initially evaluated in the emergency department of a neighboring institution after complaining of throat pain and difficulty swallowing for a duration of one week. After a benign appearing evaluation, the patient was discharged from the emergency department with prednisone, ondansetron, and ibuprofen. His symptoms persisted over the following 10 days and he decided to seek further care at our institution. On arrival, the patient complained of persistent throat pain and difficulty swallowing, left-sided ear pain, nausea, vomiting, subjective fevers, general malaise, right-sided rib pain, and decreased appetite. Initial vital signs at our institution included an oral temperature of 98.0 °F, a pulse rate of 89 beats/minute, a respiratory rate of 15 breaths/minute, and seated blood pressure of 98/50 mmHg (right arm) and 97/53 mmHg (left arm). On physical examination, marked tenderness of the left neck and right ribs was evident and the patient had difficulty opening his mouth. Laboratory studies revealed leukocytosis of 19.2 × 10^3^/uL (4.0-10.5 × 10^3^/uL), mild anemia with a hemoglobin of 13.2 g/dL (13.7-17.5 g/dL), thrombocytopenia of 11 × 10^3^/uL (163-337 × 10^3^/uL), acute kidney injury with creatinine of 1.40 mg/dL (0.43-1.13 mg/dL), hyperbilirubinemia with a total bilirubin level of 5.3 mg/dL (0.1-1.2 mg/dL), and a conjugated component of 2.8 mg/dL (0.0-0.3 mg/dL). The patient was started on dexamethasone for suspected immune thrombocytopenic purpura (ITP) and anticoagulation with apixaban was initiated. ADAMTS13 activity was later observed, showing markedly decreased activity rate of less than 2% (normal: >66.8%). Initial chest radiograph revealed scattered non-calcified pulmonary nodules (Figure 1).

**Figure 2:**
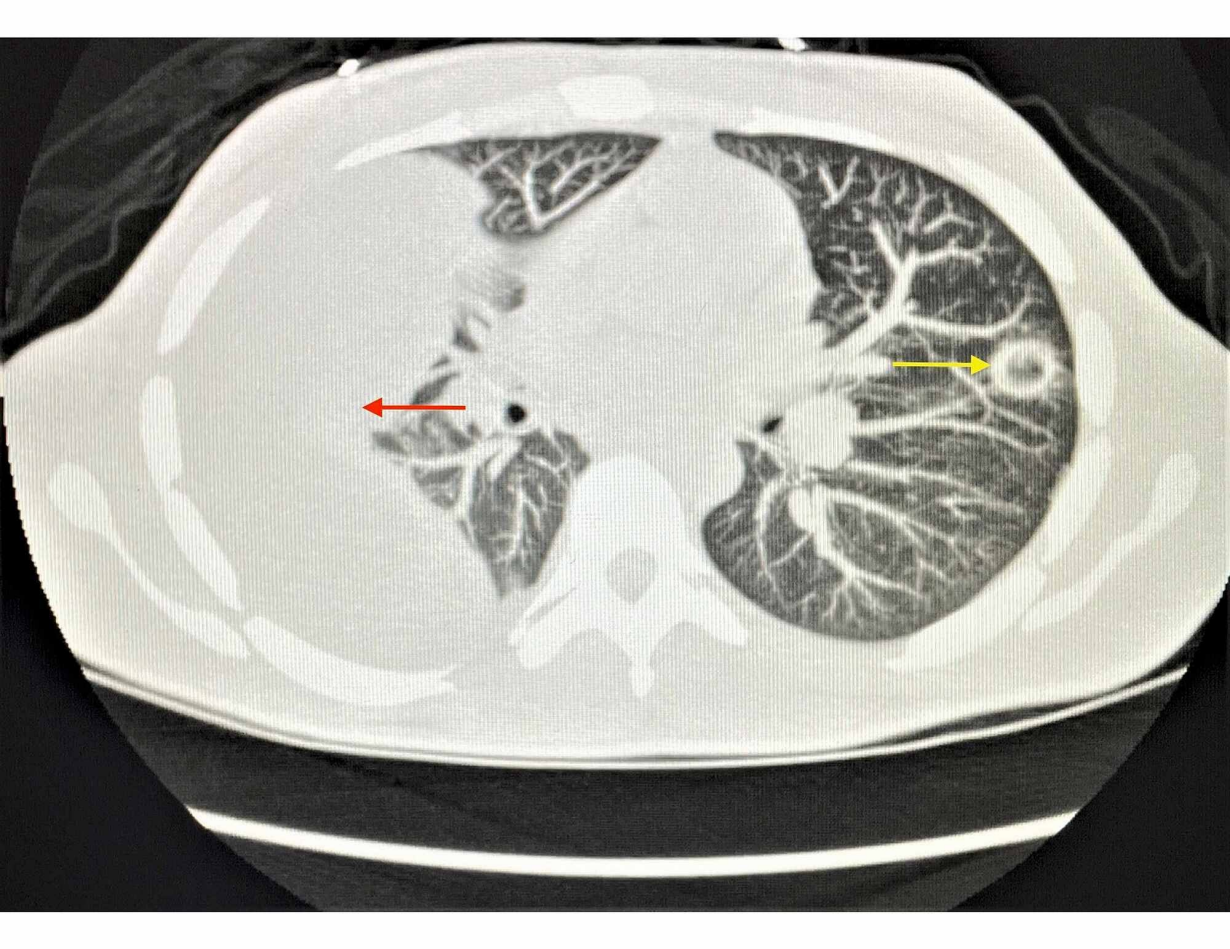
Reformatted CT chest without contrast showing right-sided loculated pleural effusion (red arrow) and left-sided cavitary lung nodule (yellow arrow) prior to pigtail catheter placement. CT, computed tomography

Computed tomography (CT) scan of the chest without contrast (Figure 2) revealed a large right-sided loculated pleural effusion in addition to numerous bilateral cavitary pulmonary nodules throughout multiple slices, which were highly concerning for septic emboli.

**Figure 3:**
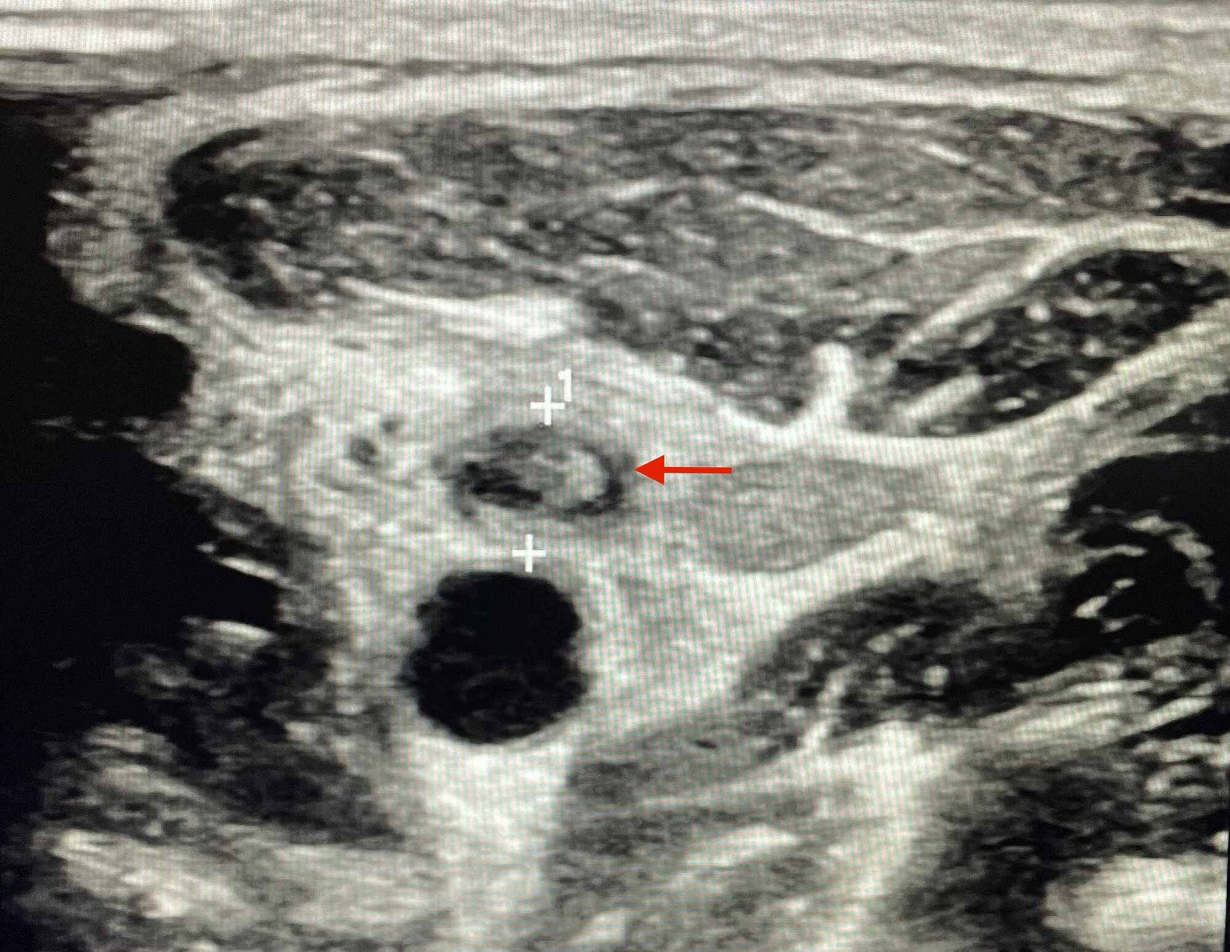
Neck ultrasound showing left internal jugular vein thrombosis (red arrow).

**Figure 4:**
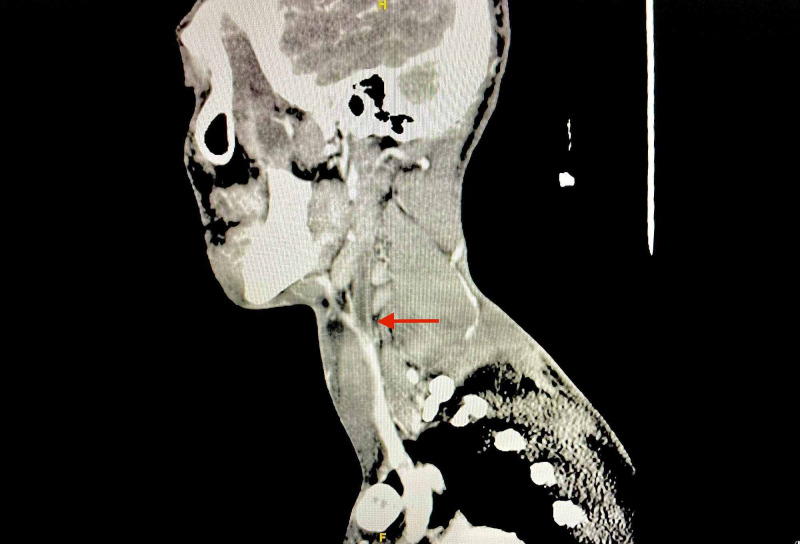
CT neck with contrast (sagittal view) showing filling defect of the left internal jugular vein (red arrow). CT, computed tomography

Neck ultrasound (Figure 3) and CT scan of neck with contrast revealed thrombosis of the left internal jugular vein (Figure 4).

**Figure 5:**
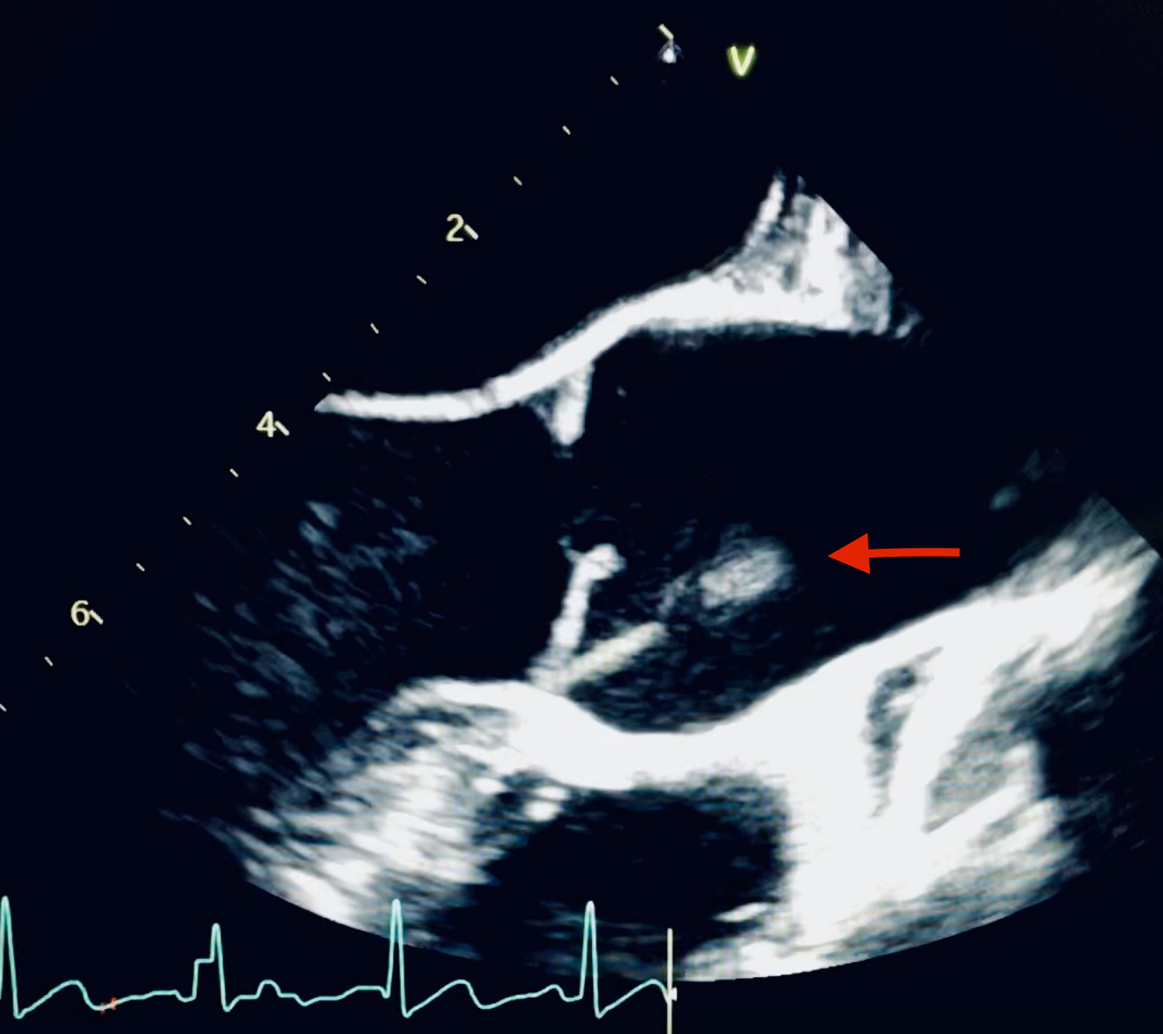
TEE showing an echodensity in the aortic root (red arrow). TEE, transesophageal echocardiogram

Cultures of blood, urine, and sputum were obtained and the patient was started on broad-spectrum antibiotic therapy with vancomycin and piperacilin-tazobactam. Blood cultures subsequently revealed *Fusobacterium necrophorum* and vancomycin was discontinued. Cardiology performed a transesophageal echocardiogram (TEE) revealing an echodensity in the aortic root possibly representing endocarditis (Figure 5).

**Figure 6:**
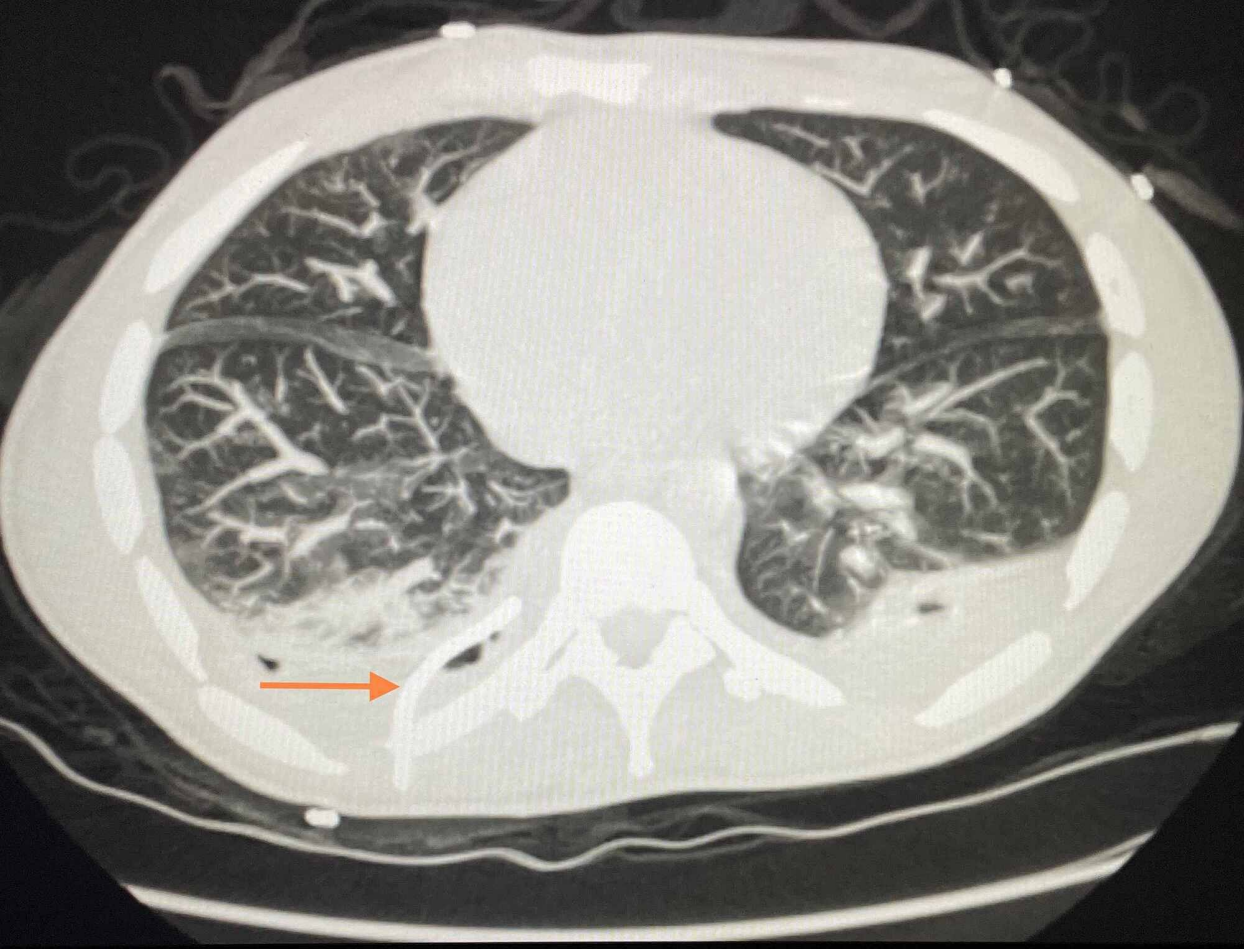
Reformatted CT chest without contrast and with pigtail catheter. Pigtail catheter (orange arrow) and resolving pleural effusion on the left following TPA administration. TPA, tissue plasminogen activator

The patient continued to complain of shortness of breath during the first two weeks of his admission despite ongoing antibiotic and anticoagulation therapy. Interval chest radiographs and CT scans were performed during the hospital course, which continued to show a large right-sided loculated pleural effusion. Interventional radiology intervened and placed a pigtail catheter with intrapleural tissue plasminogen activator (TPA) administration, eventually resulting in near resolution of the pleural effusion (Figure 6).

Following treatment, the respiratory status and vital signs of the patient improved. Routine laboratory values normalized, and the patient reported reduced pain in his neck, jaw, and ear. After a three-week hospital stay, the patient was discharged with an additional three-week course of ertapenem to complete a total of six weeks of antibiotic therapy, as well as a three-month course of anticoagulation with apixaban. The patient was followed up at the Infectious Disease and Hematology-Oncology clinic as an outpatient. After finishing antibiotic and anticoagulation therapy, repeat neck ultrasound showed resolution of the internal jugular vein thrombus, as well as normalization of his complete blood cell count and complete metabolic panel. Our patient made a full recovery with no lasting complications.

## Discussion

Lemierre’s syndrome is rarely encountered clinically. It is estimated that the yearly incidence rate of Lemierre’s syndrome is one to five cases per million people [6]. TTP shares an incidence rate with Lemierre’s syndrome, with yearly cases estimated to be 1.28-2.34 cases per million people [7]. To our knowledge, Lemierre’s syndrome accompanied by severe thrombocytopenia secondary to acquired TTP has not been described in the literature yet. The clinical course of a healthy, young male patient presenting with these two rare conditions is described in this case report.

During the hospital stay of our patient, multiple imaging studies were completed, including chest radiographs, CT scans of the head, neck, and chest, neck ultrasonography, and TEE. A full hematologic workup including a complete blood cell count with differential, coagulation profile, blood, urine, and sputum cultures, an autoimmune panel, human immunodeficiency virus, Epstein Barr virus, and hepatitis antibody testing, as well as testing for parvovirus and coronavirus were completed. Laboratory and imaging workups showed evidence of microangiopathic hemolytic anemia, a PLASMIC score of seven points, which is an important tool developed by the Harvard Thrombotic Microangiopathies (TMA) Research Collaborative to assist in the diagnosis of TTP, an ADAMTS13 level of less than 2% [1,8], multiple septic emboli in the thorax and neck, loculated pleural effusions, and positive blood cultures for *F. necrophorum*. Consulting services on the case included Infectious Disease, Hematology-Oncology, Cardiology, and Interventional Radiology. Broad-spectrum antibiotic therapy, steroids, intravenous fluids, catheter-directed TPA, analgesics, and anticoagulation were included in the management of the patient.

On hospital day 20, our patient was discharged with an additional three-week course of ertapenem for a total of six weeks of antibiotic therapy, as well as a three-month course of apixaban for anticoagulation. Although the benefits are controversial, anticoagulation has been shown to increase antibiotic penetrance into pathogenic thrombi promoting breakdown while preventing thrombi formation and local extension [9]. Due to the rare nature of Lemierre’s syndrome and TTP, the amount of data for risk-benefit analysis of anticoagulation are very limited [10]. Our patient was followed at the Infectious Disease and Hematology-Oncology clinic as an outpatient. Antibiotic and anticoagulation regimens were completed, repeat neck ultrasound displayed resolution of the internal jugular vein thrombus, and the complete blood cell count and metabolic panel values were within normal limits.

This case demonstrates that clinicians should recognize TTP as a possible cause of thrombocytopenia in patients with Lemierre’s syndrome to minimize associated morbidity and mortality of these conditions. The link between these conditions warrants further investigation as the pathogenesis of acquired TTP is not well understood.

## Conclusions

Lemierre’s syndrome is a rare but very serious medical condition that needs to be promptly diagnosed and treated. It can be complicated by severe thrombocytopenia as evidenced by our patient. While sometimes attributed to sepsis syndrome, severe thrombocytopenia in patients with Lemierre’s syndrome should prompt physicians to look for other possible causes of thrombocytopenia including TTP.
